# Combining aortic arch dissection stent implantation and root surgery for aortic dissection type A

**DOI:** 10.1186/s13019-023-02154-z

**Published:** 2023-02-10

**Authors:** Moritz Benjamin Immohr, Arash Mehdiani, Sebastian Johannes Bauer, Hayato Ise, Yukiharu Sugimura, Artur Lichtenberg, Payam Akhyari

**Affiliations:** Department of Cardiac Surgery, Medical Faculty, University Hospital Düsseldorf, Heinrich-Heine-University Düsseldorf, Moorenstrasse 5, 40225 Düsseldorf, Germany

**Keywords:** Acute aortic dissection type A, AMDS, Aortic root surgery, Dissection stent

## Abstract

**Background:**

Acute aortic dissection type A (AADA) is associated with high perioperative morbidity and mortality. A novel non-covered hybrid prosthesis (AMDS, CryoLife, Kennesaw, USA) can be easily implanted to stabilize the true lumen. However, the role of AMDS for patients requiring additional aortic root surgery has not been described.

**Methods:**

Between 2010 and 2020 a total of n = 370 patients underwent surgery for AADA in our department. Of those, n = 120 underwent treatment for aortic root in addition to proximal arch replacement without resection of the aorta beyond the innominate artery (Control, n = 111) and were compared to patients who received additional AMDS implantation (AMDS, n = 9).

**Results:**

Aortic valve repair was performed in 48.6% (Control) and in 55.6% of AMDS patients. Cardiopulmonary bypass (Control: 248 ± 76 min, AMDS: 313 ± 53 min, *P* < 0.01) time as well as circulatory arrest time of the lower body (Control: 30 ± 15 min, AMDS: 52 ± 12 min, *P* < 0.01) was prolonged in the AMDS group. Nevertheless, postoperative in-hospital morbidity such as dialysis (Control: 22.4%, AMDS: 11.1%, *P* = 0.68) and stroke (Control: 17.0%, AMDS: 22.2%, *P* = 0.65) were comparable. In-hospital death (Control: 21.8%, AMDS: 11.1%, *P* = 0.68) and the compound end-point MACCE (Control: 38.7%, AMDS: 44.4%, *P* = 0.74) did also not differ.

**Conclusions:**

Addressing the arch by implantation of AMDS prolongs cardiopulmonary bypass and circulatory arrest time, however without relevant impairments of short-term outcome. Combining root surgery with replacement of the proximal aortic arch and AMDS implantation seems feasible and safe as it did not impair the early postoperative outcome.

## Introduction

Acute aortic dissection type A (AADA) is a life-threatening disease requiring immediate emergency surgery[1, 2]. Despite multiple evolvements of the clinical management and the surgical technique, perioperative morbidity and mortality remains unsatisfyingly high[3, 4]. Controversy persists whether more or less radical procedures should be favored in the acute surgical setting[5–7]. Moreover, the strategy targeting the aortic arch is a matter of concern, especially in patients with no intima tear within the arch region [6, 8, 9]. In this context, recent development of a novel non-covered hybrid stent prosthesis (AMDS, ARTIVION, Kennesaw, USA) that can be easily implanted in the arch and descending aorta during AADA surgery has gained interest [10–13]. AMDS can stabilize the true lumen and to improve remodeling while preventing malperfusion [10–13]. However, in patients presenting with AADA complicated by relevant pathology at the level of aortic root or the aortic valve requiring surgical treatment, feasibility of concomitant AMDS implantation as a technically simplified treatment of downstream thoracic aorta has not been evaluated, yet.

We therefore retrospectively reviewed all patients treated for AADA at our center over the last decade, focusing on patients with aortic root surgery and comparing those with additional AMDS implantation with those without AMDS. Thereby, we aimed to analyze whether AMDS implantation is feasible and safe in patients who undergo combined aortic root surgery in addition to replacement of the proximal arch due to more extended forms of AADA.

## Materials and methods

### Ethics

The reported study was performed in accordance with the principles of the Declaration of Helsinki.

### Patients and study design

All patients undergoing emergency surgery for AADA between 2010 and 2020 in our department (n = 370) were retrospectively reviewed in a single center non-randomized retrospective observational cohort study design. Patient with subacute or chronic aortic dissection were excluded from the database. Detailed inclusion and exclusion criteria applied for patient selection are listed in Table 1. As a standard procedure for surgical treatment of AADA patients, all patients underwent replacement of the ascending aorta with distal anastomosis performed in circulatory arrest allowing an endoluminal inspection of the aortic arch to exclude entry sites at this region. Further extension of surgery toward the aortic root or the aortic arch was performed wherever relevant valvular pathology or extensive dissection, or tear was present at the level of the aortic root, or when an intimal tear was observed at the level of the aortic arch, respectively. A total of n = 120 patients with combined aortic root surgery and replacement of the proximal aortic arch without further treatment of the arch beyond the innominate artery and the supra-aortic vessels were identified and included. Surgery of the aortic root was defined as valve-sparing aortic root repair by the David procedure or root replacement by the Bentall procedure. Isolated use of surgical glue to realign the layers of the aortic wall was not considered as root surgery and not included. AADA patients who received concomitant AMDS implantation (n = 9, operated between August 2019 and December 2020) were compared to the remaining AADA patients without AMDS implantation (Control, n = 111, operated between January 2010 and October 2020). The excluded patients (n = 250) underwent a variety of different types of aortic surgery, including patients with isolated hemi-arch replacement without concomitant root surgery as well as total arch replacement. In patients with an intimal tear in the aortic arch, frozen elephant trunk procedure with a hybrid prosthesis was regularly performed.

**Table 1:** Inclusion and exclusion criteria

Inclusion criteria
Acute aortic dissection type A
Emergency surgery
Age > 18 years

**Exclusion criteria**
Intraoperative dissection
Subacute or chronic aortic dissection
No aortic valve surgery
Surgery of the supra-aortic vessels
Anastomosis beyond aortic arch zone 0
Isolated repair of the aortic wall by surgical glue

Inclusion and exclusion criteria for patients participating in the study

### Surgical procedure

**Fig. 1:**
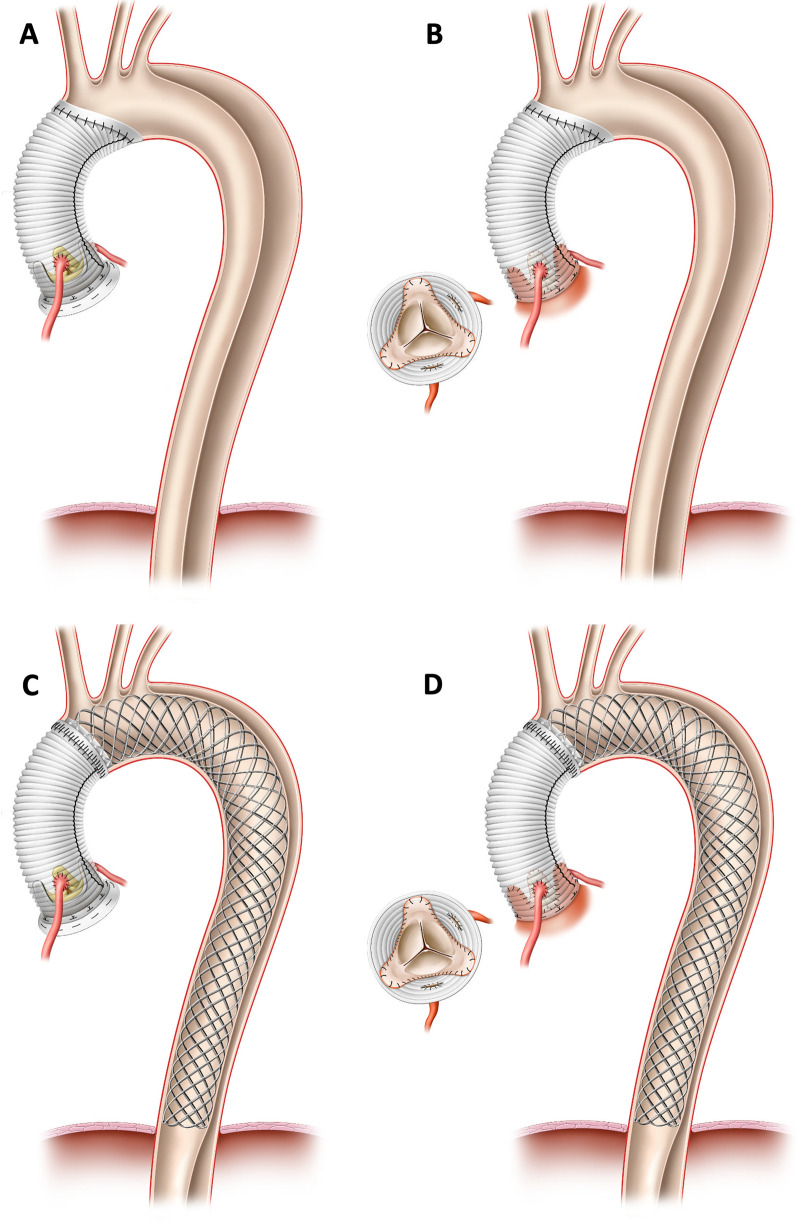
Schematic illustration of performed operative procedures. Patients with acute type A aortic dissection underwent emergency surgery of the aortic valve by either implantation of a biological or mechanical aortic valve prosthesis by the Bentall procedure (**A**, **C**) or by aortic valve repair by the David procedure (**B**, **D**) as well as implantation of a tube graft. Anastomosis was performed in aortic arch zone 0. In contrast to the control group (**A**, **B**), patients of the intervention group (**C**, **D**) received additional implantation of a novel non-covered hybrid stent prosthesis (AMDS, ARTIVION, Kennesaw, USA) in the aortic arch and descending thoracic aorta in order to stabilize the true lumen, prevent malperfusion and promote aortic remodeling. The length of the stent in the thoracic aorta varies depending on the size of the implanted prosthesis as well as the individual anatomy of the patient. Therefore, the figure only shows a schematic overview of the surgical techniques

All patients underwent emergency on-pump surgery after confirmation of AADA by contrast-enhanced computed tomography covering at least the thoracic aorta. Surgery on the aortic root included valve repair by valve spearing root replacement according to David or valve replacement. A tube graft was used for proximal arch replacement. No further treatment of the arch and the supra-aortic vessels was performed in any of the included patients. All patients underwent circulatory arrest at body core temperature of 26–28 °C to inspect the aortic arch. Near-infrared intraoperative spectroscopy (NIRS) was used to monitor cerebral oxygenation, especially during CPB time and hypothermal circulatory arrest of the lower body. Unilateral antegrade cerebral perfusion was achieved by continuing perfusion of the right subclavian artery at 8–10 ml/min/kg body weight while clamping of the innominate artery. For bilateral antegrade perfusion, an endoluminal balloon-inflatable perfusion catheter was inserted into the left common carotid artery for pressure-controlled perfusion (target perfusion pressure 60 mmHg). Bilateral antegrade cerebral perfusion was initiated directly after the beginning of circulatory arrest and the release of the aortic cross-clamp as standard technique. Rarely was an additional endoluminal perfusion catheter inserted into the left subclavian artery to increase cerebral perfusion pressure through the left vertebral artery. In the control group, the dissected native ascending aorta was resected with resection line oftentimes extended into the minor curvature of the aortic arch, resulting in classic proximal arch technique. Adventitial felt stripe were used for re-enforcement of the anastomosis line. According to the intraoperative finding and surgeons’ preference also an additional endoluminal felt stripe was used in accordance with the sandwich technique. In AMDS group the ascending aorta was resected up to the intended anastomosis line in aortic arch zone 0. AMDS was implanted in line with the manufacturer’s instructions. Anastomosis between aortic arch with AMDS and the tube graft prosthesis of the ascending aorta was sutured with a modified cuffed anastomosis technique to prevent bleeding and potential future pseudoaneurysms [14, 15]. The size of AMDS was evaluated by measuring the diameter of the aortic vessel at the level between the innominate and the left common carotid artery, as well as at the level of the tracheal bifurcation in the preoperative computed tomography-angiography scan. The recommended size of the AMDS prosthesis (40 mm straight, 55 mm straight, 40/30 mm tapered, 55/40 mm tapered) was chosen according to the manufacturer's sizing sheet. Bilateral antegrade cerebral perfusion was continued during insertion of the AMDS delivery device and while sewing the proximal felt tube of AMDS to the native aorta. Shortly before finishing the suture line, cerebral perfusion was paused, the perfusion catheter removed to allow for completion of the suture line. Afterwards, the antegrade perfusion catheter were re-inserted through individual cells of the uncovered stent body and cerebral perfusion continued while anastomosing the ascending vascular tube graft to the already implanted AMDS. Following the manufacturer’s instructions for AMDS implantation, no specific anticoagulant or antiplatelet regime after stent implantation was initiated. Figure 1 shows the performed surgical procedures of both groups.

### Primary and secondary endpoints

Incidence of major adverse cardiovascular and cerebrovascular events (MACCE) defined as postoperative stroke, in-hospital mortality and need for early re-intervention represent the combined primary endpoint of the study. ICU- and overall hospital stay, incidence of postoperative adverse events such as malperfusion, acute kidney injury or infective complications served as secondary endpoints.

### Statistics

Data analyses were performed with SPSS Statistics 28 (IBM Corporation, Armonk, NY, USA). Because of the small and unbalanced groups sizes Gaussian distribution was not assumed and variables therefore compared by either non-parametric two-tailed Mann–Whitney-U or two-tailed Fisher’s exact tests. All results are presented as mean values with the standard deviation (SD) or percentage of the whole. Statistically significant intergroup differences were defined as *P* < 0.05.

## Results

### Baseline characteristics prior to emergency surgery

Preoperative patient data including neurological status and hemodynamic is shown in Table 2. No significant differences were found between the two groups regarding demographic data as well as co-morbidities. None of the AMDS patients suffered from concomitant cardiopulmonary diseases such as coronary artery disease, diabetes or pulmonary diseases.

**Table 2:** Preoperative characteristics of the study cohort

	AMDS	Control	*P*-value
	(n = 9)	(n = 111)	
Age, y (mean ± SD)	54 ± 8	62 ± 11	0.44
Female gender, n (%)	1 (11.1)	33 (29.7)	0.44
Height, cm (mean ± SD)	178 ± 10	175 ± 10	0.57
Weight, kg (mean ± SD)	93 ± 15	83 ± 17	0.08
Body mass index, kg/m^2^ (mean ± SD)	29.3 ± 3.7	26.6 ± 3.9	0.06
Body surface area, m^2^ (mean ± SD)	2.14 ± 0.22	2.00 ± 0.24	0.09
Left ventricular ejection fraction, % (mean ± SD)	52.5 ± 5.0	52.8 ± 10.1	0.60
Co-morbidities			
Hypertension, n (%)	5 (55.6)	82 (74.5)	0.26
Diabetes, n (%)	0 (0.0)	11 (9.9)	> 0.99
Chronic kidney disease, n (%)	0 (0.0)	7 (6.3)	> 0.99
Pulmonary diseases, n (%)	0 (0.0)	11 (9.9)	> 0.99
Nicotine abuse, n (%)	5 (55.6)	36 (32.4)	0.27
Coronary artery disease, n (%)	0 (0.0)	22 (19.8)	0.21
Atrial fibrillation, n (%)	1 (11.1)	19 (17.1)	> 0.99
Previous cardiac surgery, n (%)	0 (0.0)	5 (4.5)	> 0.99
Previous cerebral infarction, n (%)	0 (0.0)	3 (2.7)	> 0.99
Marfan syndrome, n (%)	0 (0.0)	1 (0.9)	> 0.99
Acute symptoms			
Syncope, n (%)	1 (11.1)	4 (3.6)	0.35
Paresis, n (%)	1 (11.1)	15 (13.5)	> 0.99
Paresthesia, n (%)	0 (0.0)	9 (8.1)	> 0.99
Confusion, n (%)	0 (0.0)	4 (3.6)	> 0.99
Coma, n (%)	0 (0.0)	6 (5.4)	> 0.99
Acute myocardial infarction, n (%)	0 (0.0)	4 (3.6)	> 0.99
Cardiopulmonary resuscitation, n (%)	1 (11.1)	11 (9.9)	> 0.99
Preoperative mechanical ventilation, n (%)	0 (0.0)	11 (9.9)	> 0.99
Preoperative catecholamine therapy, n (%)	0 (0.0)	9 (8.1)	> 0.99
Laboratory values			
Creatinine, mg/dl (mean ± SD)	1.16 ± 0.20	1.19 ± 0.47	0.71
Urea, mg/dl (mean ± SD)	35.8 ± 7.6	43.8 ± 20.6	0.18
Creatine kinase, U/l (mean ± SD)	100 ± 59	351 ± 1569	0.58
Troponin T, ng/l (mean ± SD)	21.2 ± 22.6	253.3 ± 1502.2	0.21
Aspartate amino transferase, U/l (mean ± SD)	23.8 ± 5.6	83.9 ± 280.3	0.06
Alanine amino transferase, U/l (mean ± SD)	24.4 ± 14.3	69.2 ± 214.7	0.18
Hemoglobin, g/dl (mean ± SD)	13.6 ± 1.9	12.9 ± 2.1	0.43
Platelets, 1 × 10^3^/µl (mean ± SD)	205 ± 21	215 ± 116	0.52

Preoperative baseline characteristics prior to emergency surgery for acute type A aortic dissection. Patients with additional implantation of a novel non-covered hybrid prosthesis (AMDS, ARTIVION, Kennesaw, USA) (AMDS, n = 9) were compared to controls (n = 111). All patients received replacement of the ascending aorta by a tube graft prosthesis and aortic valve surgery. Continuous variables are presented as the mean with the standard deviation

### Operative procedures

Table 3 displays detailed information about the operative procedures. There was a moderate trend towards increased EuroSCORE II in the control patients. All patients received replacement of the ascending aorta by a tube graft prosthesis. Aortic valve repair or replacement by implantation of a bioprosthesis was performed at similar rates in both groups, in about 50% of cases, respectively. While one third of the control patients received additional coronary artery bypass grafting (CABG), not a single coronary procedure was performed in the AMDS group (*P* = 0.06). Due to the more extensive surgery by additional AMDS implantation, duration of the CPB, aortic cross-clamping and circulatory arrest time of the lower body were prolonged in this group compared to the controls (*P* < 0.01). Nevertheless, there was no difference regarding intraoperative peak lactate values as well as required blood transfusions (*P* > 0.05).

**Table 3:** Operative parameters

	AMDS	Control	*P*-value
	(n = 9)	(n = 111)	
EuroScore II, % (mean ± SD)	15.7 ± 3.7	25.4 ± 15.3	0.09
Duration			
Overall, min (mean ± SD)	471 ± 83	382 ± 103	0.09
Cardiopulmonary bypass, min (mean ± SD)	313 ± 53	248 ± 76	< 0.01
Aortic cross clamp, min (mean ± SD)	201 ± 50	149 ± 48	< 0.01
Circulatory arrest, min (mean ± SD)	52 ± 12	30 ± 15	< 0.01
Bilateral antegrade cerebral perfusion, min (mean ± SD)	38 ± 15	33 ± 45	0.03
Reperfusion, min (mean ± SD)	88 ± 22	78 ± 38	0.13
Operative procedures			
Replacement of ascending aorta, n (%)	9 (100.0)	111 (100.0)	> 0.99
Valve-sparing root replacement, n (%)	5 (55.6)	54 (48.6)	0.74
Aortic valve replacement			
Bioprosthesis, n (%)	4 (44.4)	54 (48.6)	> 0.99
Mechanical prosthesis, n (%)	0 (0.0)	3 (2.7)	> 0.99
Coronary artery bypass graft, n (%)	0 (0.0)	37 (33.3)	0.06
Arterial cannulation for cardiopulmonary bypass			
Right subclavian artery, n (%)	7 (77.8)	72 (64.9)	0.72
Ascending aorta, n (%)	2 (22.2)	22 (19.8)	> 0.99
Femoral artery, n (%)	0 (0.0)	19 (17.1)	0.35
Apex, n (%)	0 (0.0)	1 (0.9)	> 0.99
Antegrade perfusion			
Right subclavian artery, n (%)	9 (100.0)	102/106 (96.2)	> 0.99
Left common carotid artery, n (%)	9 (100.0)	101/106 (95.3)	> 0.99
Left subclavian artery, n (%)	1 (11.1)	19/106 (17.9)	> 0.99
Minimal body core temperature, °C (mean ± SD)	26.5 ± 0.8	26.6 ± 4.2	0.74
Intraoperative peak lactate, mmol/l (mean ± SD)	9.06 ± 4.00	7.93 ± 4.45	0.18
Aortic rupture, n (%)	1 (11.1)	6 (5.4)	0.43
Blood transfusion, n (%)	9 (100.0)	111 (100.0)	> 0.99
Packed red blood cells, n (mean ± SD)	9.8 ± 8.7	9.9 ± 6.1	0.49
Packed platelets, n (mean ± SD)	5.6 ± 2.5	4.9 ± 2.4	0.51
Intraoperative death, n (%)	0 (0.0)	1 (0.9)	> 0.99

Operative procedures for acute type A aortic dissection. Patients with additional implantation of a novel non-covered hybrid prosthesis (AMDS, ARTIVION, Kennesaw, USA) (AMDS, n = 9) were compared to controls (n = 111)

### Postoperative data and impact of additional AMDS implantation on primary and secondary endpoints

Postoperative in-hospital outcome is shown in Table 4. We did not observe relevant differences in the evaluated postoperative parameters between AMDS and Control group. There was one patient of the AMDS group (EuroSCORE = 19.66%) with already preoperatively existing hemiparesis and transient loss of consciousness as well as intraoperative va-ECMO implantation who suffered from postoperative multiple organ dysfunction syndrome and consecutive in-hospital death (11.1%). This patient was also diagnosed with large cerebral infarction and represents one of the two patients with stroke in the AMDS group. In contrast, n = 24 (21.8%) of the control patient did not survive the hospital stay (*P* = 0.68). Incidence of stroke (AMDS = 22.2%, control: 17.0%, *P* = 0.65) as well as reoperations (AMDS = 33.3%, control: 25.9%, *P* = 0.70) as well as MACCE (AMDS = 44.4%, control: 38.7%, *P* = 0.74) did not differ either. The same effects were also observed for kidney injury, vascular interventions and infective complications. In addition, postoperative laboratory values and blood transfusions were comparable.

**Table 4:** Postoperative outcome

	AMDS	Control	*P*-value
	(n = 9)	(n = 111)	
Hospital stay, d (mean ± SD)	20.4 ± 19.9	17.0 ± 13.3	0.87
ICU stay, h (mean ± SD)	140 ± 190	186 ± 272	0.51
Mechanical ventilation, h (mean ± SD)	23.3 ± 17.9	50.3 ± 54.0	0.27
Tracheostomy, n (%)	1 (11.1)	9/106 (8.5)	0.57
Chest tube drainage volume, ml (mean ± SD)	901 ± 617	1392 ± 1144	0.13
Blood transfusions			
Packed red blood cells, n (mean ± SD)	1.78 ± 2.11	2.72 ± 4.43	0.87
Packed platelets, n (mean ± SD)	0.67 ± 0.87	0.90 ± 2.04	0.52
Packed fresh frozen plasma, n (mean ± SD)	3.56 ± 4.13	3.50 ± 4.57	0.86
Left ventricular ejection fraction, % (mean ± SD)	45.8 ± 22.7	46.8 ± 24.5	0.34
In-hospital adverse events			
MACCE, n (%)	4 (44.4)	43/111 (38.7)	0.74
In-hospital death, n (%)	1 (11.1)	24/110 (21.8)	0.68
Death within 24 h after surgery, n (%)	0 (0.0)	10/110 (9.1)	> 0.99
Extracorporeal life support, n (%)	1 (11.1)	13/106 (12.3)	> 0.99
Cardiopulmonary resuscitation, n (%)	2 (22.2)	10/105 (9.5)	0.24
Reoperation, n (%)	3 (33.3)	28/108 (25.9)	0.70
Thoracic bleeding, n (%)	2 (66.7)	23 (82.1)	
Pacemaker implantation, n (%)	0 (0.0)	1 (3.6)	
Wound healing disorder, n (%)	0 (0.0)	1 (3.6)	
Other, n (%)	1 (33.3)	3 (10.7)	
Vascular intervention, n (%)	1 (11.1)	13/106 (12.3)	> 0.99
Multi organ dysfunction syndrome, n (%)	1 (11.1)	6/106 (5.7)	0.44
Stroke, n (%)	2 (22.2)	18/106 (17.0)	0.65
Thromboembolism, n (%)	1 (11.1)	4/106 (3.8)	0.34
Hemodialysis, n (%)	1 (11.1)	24/107 (22.4)	0.68
Sepsis, n (%)	0 (0.0)	1/106 (0.9)	> 0.99
Compartment syndrome, n (%)	0 (0.0)	4/105 (3.8)	> 0.99
Hematothorax, n (%)	2 (22.2)	18/108 (16.7)	0.65
Arrhythmia, n (%)	5 (55.6)	48/108 (44.4)	0.73
Respiratory failure, n (%)	2 (22.2)	20/107 (18.7)	0.68
Postoperative laboratory values			
First postoperative day			
Troponin T, ng/l (mean ± SD)	11,649 ± 30,155	3,328 ± 6,378	0.95
Creatine kinase, U/l (mean ± SD)	3,351 ± 3,761	5,385 ± 16,518	0.39
Creatinine, mg/dl (mean ± SD)	1.48 ± 0.41	1.68 ± 0.74	0.80
Urea, mg/dl (mean ± SD)	45.6 ± 11.9	65.5 ± 30.4	0.03
Hemoglobin, g/dl (mean ± SD)	9.43 ± 1.80	9.53 ± 1.71	0.50
Aspartate amino transferase, U/l (mean ± SD)	283 ± 259	348 ± 581	0.44
Alanine amino transferase, U/l (mean ± SD)	123 ± 96	141 ± 245	0.30
Platelets, 10^3^/µl (mean ± SD)	130 ± 35	140 ± 75	0.97
Fifth postoperative day			
Troponin T, ng/l (mean ± SD)	522 ± 239	1,459 ± 1,989	0.23
Creatine kinase, U/l (mean ± SD)	924 ± 930	1,689 ± 6,023	0.38
Creatinine, mg/dl (mean ± SD)	1.27 ± 0.57	1.40 ± 0.79	0.86
Urea, mg/dl (mean ± SD)	84.6 ± 37.2	69.3 ± 33.2	0.24
Hemoglobin, g/dl (mean ± SD)	9.24 ± 0.47	10.06 ± 1.46	0.08
Aspartate amino transferase, U/l (mean ± SD)	647 ± 1,330	137 ± 285	0.72
Alanine amino transferase, U/l (mean ± SD)	788 ± 869	98 ± 107	< 0.01
Platelets, 10^3^/µl (mean ± SD)	131 ± 44	138 ± 75	0.97

Postoperative in-hospital outcome after surgery for acute type A aortic dissection. Patients with additional implantation of a novel non-covered hybrid prosthesis (AMDS, ARTIVION, Kennesaw, USA) (AMDS, n = 9) were compared to controls (n = 111)

*ICU* intensive care unit, *MACCE* major adverse cardiovascular and cerebrovascular event

## Discussion

We aimed at evaluating the safety and feasibility of additional AMDS implantation in patients who need additional aortic valve or root surgery in addition to the standard replacement of the proximal arch for AADA. We were able to compare our study group consisting of patients with root surgery and additional AMDS implantation to a comparable control group receiving valve or root surgery and proximal arch surgery with no further treatment of the downstream thoracic aorta. Although AMDS implantation prolonged the operative procedure, it did not impair the short-term outcome, suggesting the initial hypothesis of feasibility and safety of this approach for patients who are already in need of extended surgery addressing the aortic root in the acute setting of AADA.

Pre-operative baseline parameters showed little differences between the AMDS and the control group minimizing potential biases. However, there was a trend towards increased EuroScore II values in the control group, which goes in line with the observed increased necessity of concomitant CABG [16]. Zhang and colleagues have reported increased early postoperative mortality in AADA patients with concomitant CABG procedures[17]. In fact, CABG is indicated in patients with coronary malperfusion due to AADA affecting the coronary ostium [18]. Therefore, CABG was regularly performed in our series for patients suffering from this condition. However, most likely due to the small group size, it has not been observed in the AMDS group. In addition, it has also not been described in AMDS patients in the currently available literature either. Though, as AMDS implantation affects only the arch and the proximal descending aorta, effects on coronary perfusion seemed to be unlikely [12, 13].

Previous studies report a median implantation time of AMDS of less than five minutes [10, 12]. In our study, circulatory arrest time of the lower body was about 20 min prolonged compared to the control group. This was caused by two reasons: First, in contrast to previously reported studies, we enforced the anastomosis line of the AMDS with an additional continuous suture line. Furthermore, we used a cuffed anastomosis technique between the tube graft prostheses and the native aorta in the AMDS group [14, 15]. These technical details may have contributed to the prolonged times in this series. However, the actual employment time of the AMDS graft was comparable with the previously reported data [10, 12].

Circulatory arrest time is associated with impaired neurological outcome after AADA surgery [19]. Nevertheless, antegrade cerebral perfusion enables to securely expand this period to more than one hour without increasing the perioperative risk [20]. Mean lower body circulatory arrest time was 52 min in the AMDS and 30 min in the Control group. Subsequently, we did not observe differences in the perioperative adverse events, in particular there was no increase in the stroke rate the AMDS group although the circulatory arrest times was prolonged in comparison to the Control group.

AMDS was originally invented to stabilize the true lumen of the arch and downstream aorta and to prevent malperfusion and the largest so far reported cohort from an international, multicenter registry including 47 patients confirms the potential benefit of AMDS in the clinical setting [10]. However, focused evaluation of combined procedures for AADA with concomitant AMDS implantation is still missing. Montagner and colleagues described a heterogeneous cohort of 16 AMDS patients [13]. Of those, two patients underwent David procedure, and five underwent patients aortic valve replacement [13]. Although there was no control group in this study, the reported results compare well with our findings [13]. Therefore, concomitant AMDS implantation in AADA patients with aortic valve surgery and proximal arch replacement seems feasible with good short-term results. These results appear promising, as published early and mid-term follow-up data suggest that patients may benefit from the additional treatment of the downstream thoracic aorta by AMDS implantation [10, 12, 13]. However, as reliable long-term data is not available yet, the potential effects remain unclear. Especially, a previous dissection stent device, the Djumbodis prosthesis (Saint Côme-Chirurgie, Marseille, France), failed to reduce false lumen patency and was associated with few late complications such as device deformity [21, 22].

The current study is limited by the still small and unbalanced group sizes with differences regarding date of the operation, EuroSCORE II, concomitant CABG and circulatory arrest time. In addition, the retrospective character of the study further decreases its power and the information on preoperative organ malperfusion is limited to preoperative neurological and hemodynamic impairments, as well as postoperative organ dysfunctions, as an indicator of persistent postoperative malperfusion. Finally, currently we are only able to present short-term data to demonstrate the feasibility and safety of combining root surgery with AMDS implantation. However, long-term effects still need to be validated in our cohort.

## Conclusion

Therapy of AADA remains challenging. Addressing the arch and downstream thoracic aorta by implantation of AMDS in cases needing aortic valve surgery prolongs cardiopulmonary bypass and circulatory arrest time, without relevant impairment of short-term outcome. Therefore, combining aortic root surgery, replacement of the ascending aorta and proximal aortic arch replacement with AMDS implantation seems feasible and safe and offers a novel therapy approach.

## Data Availability

The data underlying this article will be shared on reasonable re-quest to the corresponding author.
